# Supplementary material for: Spatiotemporal Patterns of Menin Localization in Developing Murine Brain: Co-Expression with the Elements of Cholinergic Synaptic Machinery
Source: Cells. 2021 May 16;10(5):1215. doi: 10.3390/cells10051215 (PMC8156519; doi:10.3390/cells10051215)
Supplement: Supplementary file 1 [file cells-10-01215-s001.zip › cells-1202558-supplementary.pdf]

Supplementary Table S1. SP8 Leica (confocal) and Olympus VS120 fluorescent microscope settings. (Hotchkiss Brain Institute Advanced Microscopy Platform: HBIAMP, University of Calgary, AB, Canada.

**Fluorescent microscope settings for data presented in:**	**SP8 Leica LIA - Laser Wavelength**	**Laser Power**
Figure 1A, C<br>Figure 2A<br>Figure 3A<br>Figure 4A<br>Figure 6<br>Figure 7<br>Figure 8	402.7	2.5
	568.1	5.3
	680.0	4.5
Figure 1B<br>Figure 4B<br>Figure 5	402.7	2.5
	568.1	4.2
	680.0	4
**Fluorescent microscope settings for data presented in:**	**Olympus VS120 fluorescence filters**	**Exposure time**
Figure 1–8	TRITC	100ms
	DAPI	50ms
	TRITC	100ms
	Cy5	75ms

Supplementary Table S2. Related to Figure 1D. Temporal Statistical analysis of qPCR analysis of Multiple endocrine neoplasia type 1, *MEN1* gene in E16, E18, P10 and Adult mouse whole brain slices.

**Ages**	**Mean Diff.**	**95.00% CI of diff.**	**Significant?**	**Summary**	**Adjusted *p* Value**
E16 vs. E18	−0.0003063	-0.0006463 to 3.368e-005	No	ns	0.0657
E16 vs. P10	−0.005047	-0.01388 to 0.003786	No	ns	0.1929
E16 vs. Adult	−1.911	-3.015 to -0.8080	Yes	*	0.0109
E18 vs. P10	−0.004741	-0.01385 to 0.004369	No	ns	0.2337
E18 vs. Adult	−1.911	-3.014 to -0.8078	Yes	*	0.0109
P10 vs. Adult	−1.906	-3.016 to -0.7971	Yes	*	0.0111

One-way ANOVA followed by Post-hoc-Tukey's multiple comparison test; $n = 6$, 3 independent experiments each, triplicate replicates;* $p < 0.01$, ns ≥ 0.05

Supplementary Table S3. Related to Figure 3. Mean comparison of degree of colocalization of NFL with menin between different aged mouse brain (temporal).

	E16	E18	P10	Adult
t, df	t = 7.821, df = 9	t = 8.162, df = 9	t = 12.88, df = 9	t = 9.362, df = 9
P value (two tailed)	<0.0001	<0.0001	<0.0001	<0.0001
P value summary	****	****	****	****
Significant (alpha=0.05)?	Yes	Yes	Yes	Yes

One sample t-test; n refers to the analysis of NFL and menin D.O.C. between different ages of mouse brain, ROIs derived from 14 separate images from 13 independent experiments; **** $p < 0.0001$

Supplementary Table S4. Related to Figure 4. Mean comparison of degree of colocalization of astrocytes with menin between different aged mouse brain (temporal).

One sample t test	E16	E18	P10	Adult
t, df	t = 13.07, df = 9	t = 12.37, df = 9	t = 4.152, df=9	t = 0.9990, df = 9
P value (two tailed)	<0.0001	<0.0001	0.0025	0.3439
P value summary	****	****	**	ns
Significant (alpha=0.05)?	Yes	Yes	Yes	No

One sample t-test; *n* refers to the analysis of astrocytes and menin D.O.C. between different ages of mouse brain, ROIs derived from 14 separate images from 13 independent experiments; ** $p < 0.01$, **** $p < 0.0001$, ns ≥ 0.05

Supplementary Table S5. Related to Figure 5. Statistical analysis of IHC fluorescence of menin in different aged (temporal) mouse brain slices.

**Tukey's mc test**	**Mean Diff.**	**95.00% CI of diff.**	**Significant?**	**Summary**	**Adjusted *p* Value**
E12.5 vs. E15	–12.77	–15.95 to -9.601	Yes	****	<0.0001
E12.5 vs. E18	–20.49	–23.66 to -17.32	Yes	****	<0.0001
E12.5 vs. P10	–22.82	–25.99 to -19.65	Yes	****	<0.0001
E12.5 vs. Adult	–26.92	–30.09 to -23.74	Yes	****	<0.0001
E15 vs. E18	–7.714	–10.89 to -4.541	Yes	****	<0.0001
E15 vs. P10	–10.05	–13.22 to -6.875	Yes	****	<0.0001
E15 vs. Adult	–14.14	–17.32 to -10.97	Yes	****	<0.0001
E18 vs. P10	–2.333	–5.506 to 0.8396	No	ns	0.2329
E18 vs. Adult	–6.429	–9.602 to -3.256	Yes	****	<0.0001
P10 vs. Adult	–4.095	–7.268 to -0.9223	Yes	**	0.0064

One-way ANOVA followed by Post-hoc-Tukey's multiple comparison test; *n* refers to the analysis of menin C-terminal epitope fluorescence intensity within whole brain slices, ROIs derived from ≥ 13 separate images from 7 independent experiments in all age groups; ** $p < 0.01$, **** $p < 0.0001$, ns ≥ 0.05

Supplementary Table S6a. Related to Figure 6. Statistical analysis of IHC fluorescence of menin in E12.5 mouse whole brain slices.

**Regions**	**Mean Diff.**	**95.00% CI of diff.**	**Significant?**	**Summary**	**Adjusted *p* Value**
Di vs. Te	2.659	0.4649 to 4.854	Yes	*	0.0122
Di vs. Mt	5.792	3.598 to 7.986	Yes	****	<0.0001
Di vs. Ms	13.14	10.89 to 15.39	Yes	****	<0.0001
Te vs. Mt	3.133	0.9383 to 5.327	Yes	**	0.0025
Te vs. Ms	10.48	8.232 to 12.73	Yes	****	<0.0001
Mt vs. Ms	7.348	5.099 to 9.596	Yes	****	<0.0001

One-way ANOVA followed by Post-hoc-Tukey's multiple comparison test; *n* refers to the analysis of menin C-terminal epitope fluorescence intensity within whole brain slices, ROIs derived from 13 separate images from 7 independent experiments; * $p < 0.01$, ** $p < 0.01$, **** $p < 0.0001$

Supplementary Table S6b. Related to Figure 6. IHC fluorescence of menin in E12.5 mouse brain slices.

**Fluorescence Intensity**	**Di**	**Te**	**Mt**	**Ms**
**Mean**	18.38	15.72	12.59	5.239
**Std. Error of Mean**	0.5899	0.7397	0.4685	0.4811

Supplementary Table S7a. Related to Figure 6. Statistical analysis of IHC fluorescence of menin in E15.5 mouse whole brain slices.

**Regions**	**Mean Diff.**	**95.00% CI of diff.**	**Significant?**	**Summary**	**Adjusted *p* Value**
Di vs. Te	–3.772	–6.069 to –1.474	Yes	***	0.0004
Di vs. Mt	4.744	2.447 to 7.041	Yes	****	<0.0001
Di vs. Ms	13.58	11.23 to 15.94	Yes	****	<0.0001
Te vs. Mt	8.515	6.218 to 10.81	Yes	****	<0.0001
Te vs. Ms	17.35	15.00 to 19.71	Yes	****	<0.0001
Mt vs. Ms	8.839	6.485 to 11.19	Yes	****	<0.0001

One-way ANOVA followed by Post-hoc-Tukey's multiple comparison test; *n* refers to the analysis of menin C-terminal epitope fluorescence intensity within whole brain slices, ROIs derived from 13 separate images from 7 independent experiments; *** $p < 0.001$, **** $p < 0.0001$

Supplementary Table S7b. Related to Figure 6. IHC fluorescence of menin in E15.5 mouse brain slices.

**Fluorescence Intensity**	**Di**	**Te**	**Mt**	**Ms**
**Mean**	20.34	24.11	15.59	6.755
**Std. Error of Mean**	0.3336	0.7853	0.6802	0.5397

Supplementary Table S8a. Related to Figure 6. Statistical analysis of IHC fluorescence of menin in E18 mouse whole brain slices.

**Regions**	**Mean Diff.**	**95.00% CI of diff.**	**Significant?**	**Summary**	**Adjusted *p* Value**
Co vs. Hc	–3.639	–6.783 to –0.4954	Yes	*	0.0157
Co vs. Th	–8	–11.14 to –4.857	Yes	****	<0.0001
Co vs. Ce	4.282	1.139 to 7.426	Yes	**	0.0029
Co vs. Ht	6.779	3.555 to 10.00	Yes	****	<0.0001
Hc vs. Th	–4.361	–7.573 to –1.150	Yes	**	0.003
Hc vs. Ce	7.921	4.710 to 11.13	Yes	****	<0.0001
Hc vs. Ht	10.42	7.128 to 13.71	Yes	****	<0.0001
Th vs. Ce	12.28	9.071 to 15.49	Yes	****	<0.0001
Th vs. Ht	14.78	11.49 to 18.07	Yes	****	<0.0001
Ce vs. Ht	2.497	–0.7937 to 5.788	No	ns	0.2168

One-way ANOVA followed by Post-hoc-Tukey's multiple comparison test; *n* refers to the analysis of menin C-terminal epitope fluorescence intensity within whole brain slices, ROIs derived from 13 separate images from 7 independent experiments; * $p < 0.1$,** $p < 0.01$, **** $p < 0.0001$, ns ≥ 0.05

Supplementary Table S8b. Related to Figure 6. IHC fluorescence of menin in E18 mouse brain slices.

**Fluorescence Intensity**	**Co**	**Hc**	**Th**	**Ce**	**Ht**
**Mean**	26.53	30.17	34.53	22.25	19.75
**Std. Error of Mean**	0.5477	0.703	0.8735	0.9065	0.9766

Supplementary Table S9a. Related to Figure 6. Statistical analysis of IHC fluorescence of menin in P10 mouse whole brain slices.

**Regions**	**Mean Diff.**	**95.00% CI of diff.**	**Significant?**	**Summary**	**Adjusted *p* Value**
Co vs. Hc	–4.338	–8.326 to –0.3490	Yes	*	0.0268
Co vs. Th	–9.537	–13.53 to –5.548	Yes	****	<0.0001
Co vs. Ce	2.382	-1.607 to 6.371	No	ns	0.4493
Co vs. Ht	5.35	1.259 to 9.442	Yes	**	0.0047
Hc vs. Th	–5.199	–9.274 to -1.125	Yes	**	0.0061
Hc vs. Ce	6.72	2.645 to 10.79	Yes	***	0.0002
Hc vs. Ht	9.688	5.513 to 13.86	Yes	****	<0.0001

Th vs. Ce	11.92	7.844 to 15.99	Yes	****	<0.0001
Th vs. Ht	14.89	10.71 to 19.06	Yes	****	<0.0001
Ce vs. Ht	2.968	–1.207 to 7.143	No	ns	0.2754

One-way ANOVA followed by Post-hoc-Tukey's multiple comparison test; *n* refers to the analysis of menin C-terminal epitope fluorescence intensity within whole brain slices, ROIs derived from 13 separate images from 7 independent experiments; * $p < 0.1$, ** $p < 0.01$, *** $p < 0.001$, **** $p < 0.0001$, ns ≥ 0.05

Supplementary Table S9b. Related to Figure 6. IHC fluorescence of menin in P10 mouse brain slices.

**Fluorescence Intensity**	**Co**	**Hc**	**Th**	**Ce**	**Ht**
**Mean**	27.75	32.09	37.29	25.37	22.4
**Std. Error of Mean**	1.238	1.43	0.6834	0.8068	0.4705

Supplementary Table S10a. Related to Figure 6. Statistical analysis of IHC fluorescence of menin in Adult mouse whole brain slices.

**Regions**	**Mean Diff.**	**95.00% CI of diff.**	**Significant?**	**Summary**	**Adjusted *p* Value**
Cortex vs. Hippocampus	–15.60	–18.68 to –12.52	Yes	****	<0.0001
Cortex vs. Thalamus	–24.29	–27.37 to –21.21	Yes	****	<0.0001
Cortex vs. Cerebellum	6.489	3.410 to 9.569	Yes	****	<0.0001
Cortex vs. Hypothalamus	8.561	5.403 to 11.72	Yes	****	<0.0001
Cortex vs. Pons	17.94	14.78 to 21.10	Yes	****	<0.0001
Cortex vs. medulla	19.15	15.99 to 22.31	Yes	****	<0.0001
Cortex vs. midbrain	13.57	10.42 to 16.73	Yes	****	<0.0001
Cortex vs. olfactory bulb	15.19	12.03 to 18.34	Yes	****	<0.0001
Cortex vs. caudate putamen	17.06	13.91 to 20.22	Yes	****	<0.0001
Cortex vs. basal forebrain	14.16	11.00 to 17.32	Yes	****	<0.0001
Cortex vs. ventral striatum	12.11	8.948 to 15.26	Yes	****	<0.0001
Hippocampus vs. Thalamus	–8.690	–11.84 to –5.545	Yes	****	<0.0001
Hippocampus vs. Cerebellum	22.09	18.94 to 25.23	Yes	****	<0.0001
Hippocampus vs. Hypothalamus	24.16	20.94 to 27.38	Yes	****	<0.0001
Hippocampus vs. Pons	33.54	30.31 to 36.76	Yes	****	<0.0001
Hippocampus vs. medulla	34.75	31.52 to 37.97	Yes	****	<0.0001
Hippocampus vs. midbrain	29.17	25.95 to 32.40	Yes	****	<0.0001
Hippocampus vs. olfactory bulb	30.78	27.56 to 34.01	Yes	****	<0.0001

Hippocampus vs. caudate putamen	32.66	29.44 to 35.89	Yes	****	<0.0001
Hippocampus vs. basal forebrain	29.76	26.53 to 32.98	Yes	****	<0.0001
Hippocampus vs. ventral striatum	27.70	24.48 to 30.93	Yes	****	<0.0001
Thalamus vs. Cerebellum	30.78	27.63 to 33.92	Yes	****	<0.0001
Thalamus vs. Hypothalamus	32.85	29.63 to 36.07	Yes	****	<0.0001
Thalamus vs. Pons	42.23	39.00 to 45.45	Yes	****	<0.0001
Thalamus vs. medulla	43.44	40.21 to 46.66	Yes	****	<0.0001
Thalamus vs. midbrain	37.86	34.64 to 41.09	Yes	****	<0.0001
Thalamus vs. olfactory bulb	39.47	36.25 to 42.70	Yes	****	<0.0001
Thalamus vs. caudate putamen	41.35	38.13 to 44.58	Yes	****	<0.0001
Thalamus vs. basal forebrain	38.45	35.22 to 41.67	Yes	****	<0.0001
Thalamus vs. ventral striatum	36.39	33.17 to 39.62	Yes	****	<0.0001
Cerebellum vs. Hypothalamus	2.072	−1.151 to 5.295	No	ns	0.5916
Cerebellum vs. Pons	11.45	8.225 to 14.67	Yes	****	<0.0001
Cerebellum vs. medulla	12.66	9.436 to 15.88	Yes	****	<0.0001
Cerebellum vs. midbrain	7.086	3.862 to 10.31	Yes	****	<0.0001
Cerebellum vs. olfactory bulb	8.697	5.473 to 11.92	Yes	****	<0.0001
Cerebellum vs. caudate putamen	10.57	7.351 to 13.80	Yes	****	<0.0001
Cerebellum vs. basal forebrain	7.670	4.446 to 10.89	Yes	****	<0.0001
Cerebellum vs. ventral striatum	5.617	2.394 to 8.840	Yes	****	<0.0001
Hypothalamus vs. Pons	9.376	6.077 to 12.68	Yes	****	<0.0001
Hypothalamus vs. medulla	10.59	7.288 to 13.89	Yes	****	<0.0001
Hypothalamus vs. midbrain	5.013	1.714 to 8.312	Yes	****	<0.0001

Hypothalamus vs. olfactory bulb	6.624	3.325 to 9.923	Yes	****	<0.0001
Hypothalamus vs. caudate putamen	8.502	5.203 to 11.80	Yes	****	<0.0001
Hypothalamus vs. basal fore-brain	5.597	2.298 to 8.896	Yes	****	<0.0001
Hypothalamus vs. ventral stri-atum	3.545	0.2460 to 6.844	Yes	*	0.0238
Pons vs. me-dulla	1.211	–2.088 to 4.510	No	ns	0.9858
Pons vs. mid-brain	–4.363	–7.662 to -1.064	Yes	**	0.0014
Pons vs. olfac-tory bulb	–2.752	–6.051 to 0.5470	No	ns	0.2011
Pons vs. cau-date putamen	–0.8740	–4.173 to 2.425	No	ns	0.9992
Pons vs. basal forebrain	–3.779	–7.078 to –0.4800	Yes	*	0.0111
Pons vs. ven-tral striatum	–5.831	–9.130 to –2.532	Yes	****	<0.0001
medulla vs. midbrain	–5.574	–8.873 to –2.275	Yes	****	<0.0001
medulla vs. ol-factory bulb	–3.963	–7.262 to –0.6640	Yes	**	0.0059
medulla vs. caudate puta-men	–2.085	–5.384 to 1.214	No	ns	0.6172
medulla vs. basal forebrain	–4.990	–8.289 to –1.691	Yes	***	0.0001
medulla vs. ventral stria-tum	–7.042	–10.34 to –3.743	Yes	****	<0.0001
midbrain vs. olfactory bulb	1.611	–1.688 to 4.910	No	ns	0.8943
midbrain vs. caudate puta-men	3.489	0.1900 to 6.788	Yes	*	0.0284
midbrain vs. basal forebrain	0.5840	–2.715 to 3.883	No	ns	>0.9999
midbrain vs. ventral stria-tum	–1.468	–4.767 to 1.831	No	ns	0.9416
olfactory bulb vs. caudate putamen	1.878	–1.421 to 5.177	No	ns	0.7567
olfactory bulb vs. basal fore-brain	–1.027	–4.326 to 2.272	No	ns	0.9964
olfactory bulb vs. ventral stri-atum	–3.079	–6.378 to 0.2196	No	ns	0.0915
caudate puta-men vs. basal forebrain	–2.905	–6.204 to 0.3940	No	ns	0.1416

caudate putamen vs. ventral striatum	–4.957	–8.256 to –1.658	Yes	***	0.0001
basal forebrain vs. ventral striatum	–2.052	–5.351 to 1.247	No	ns	0.6402

One-way ANOVA followed by Post-hoc-Tukey's multiple comparison test; *n* refers to the analysis of menin C-terminal epitope fluorescence intensity within whole brain slices, ROIs derived from 13 separate images from 7 independent experiments; * $p < 0.1$, ** $p < 0.01$, *** $p < 0.001$, **** $p < 0.0001$, ns ≥ 0.05

Supplementary Table S10b. Related to Figure 6. IHC fluorescence of menin in Adult mouse brain slices.

Fluorescence Intensity	Co	Hc	Th	Ce	Ht
**Mean**	32.83	48.43	57.12	26.34	24.27
**Std. Error of Mean**	0.7364	1.121	0.6776	0.6695	0.4603

Supplementary Table S11a. Related to Figure 7. Mean comparison of degree of colocalization of PSD-95 with menin between different aged mouse brain slices.

Ages	Mean Diff.	95.00% CI of diff.	Significant?	Summary	Adjusted *p* Value
E15 vs. E18	–0.024	–0.08336 to 0.03536	No	ns	0.6985
E15 vs. P10	0.168	0.1086 to 0.2274	Yes	****	<0.0001
E15 vs. Adult	–0.008	–0.06736 to 0.05136	No	ns	0.9834
E18 vs. P10	0.192	0.1326 to 0.2514	Yes	****	<0.0001
E18 vs. Adult	0.016	–0.04336 to 0.07536	No	ns	0.8861
P10 vs. Adult	–0.176	–0.2354 to -0.1166	Yes	****	<0.0001

One-way ANOVA followed by Post-hoc-Tukey's multiple comparison test; *n* refers to the analysis of PSD-95 and menin D.O.C. within different aged whole brain slices, ROIs derived from 13 separate images from 7 independent experiments; **** $p < 0.0001$, ns ≥ 0.05

Supplementary Table S11b. Related to Figure 7. Mean of degree of colocalization of PSD-95 with menin in different aged mouse brain slices.

Degree of colocalization	E15	E18	P10	Adult
**Mean**	0.812	0.836	0.644	0.82
**Std. Error of Mean**	0.01031	0.009684	0.02583	0.01022

Supplementary Table S12a. Related to Figure 7. Mean comparison of degree of colocalization of SYT with menin between different aged mouse brain slices.

Ages	Mean Diff.	95.00% CI of diff.	Significant?	Summary	Adjusted P Value
E15 vs. E18	–0.0558	-0.08391 to -0.02769	Yes	****	<0.0001
E15 vs. P10	0.0742	0.04609 to 0.1023	Yes	****	<0.0001
E15 vs. Adult	–0.1608	-0.1889 to -0.1327	Yes	****	<0.0001
E18 vs. P10	0.13	0.1019 to 0.1581	Yes	****	<0.0001
E18 vs. Adult	–0.105	–0.1331 to –0.07689	Yes	****	<0.0001
P10 vs. Adult	–0.235	–0.2631 to –0.2069	Yes	****	<0.0001

One-way ANOVA followed by Post-hoc-Tukey's multiple comparison test; *n* refers to the analysis of SYT and menin D.O.C. within different aged whole brain slices, ROIs derived from 13 separate images from 7 independent experiments; **** $p < 0.0001$

Supplementary Table S12b. Related to Figure 7. Mean of degree of colocalization of SYT with menin in different aged mouse brain slices.

Degree of colocalization	E15	E18	P10	Adult
**Mean**	0.7352	0.791	0.661	0.896
**Std. Error of Mean**	0.006987	0.006046	0.006403	0.009568

Supplementary Table S13a. Related to Figure 8. Mean comparison of degree of colocalization of PSD-95 with menin between different Days in-vitro (DIV) in hippocampal neuronal cultures.

DIV	Mean Diff.	95.00% CI of diff.	Significant?	Summary	Adjusted *p* Value
DIV4 vs. DIV7	0.007778	–0.05845 to 0.07401	No	ns	0.9886
DIV4 vs. DIV10	–0.01656	–0.08279 to 0.04968	No	ns	0.9049
DIV4 vs. DIV12	–0.04422	–0.1105 to 0.02201	No	ns	0.2878
DIV7 vs. DIV10	–0.02433	–0.09056 to 0.04190	No	ns	0.7531
DIV7 vs. DIV12	–0.052	–0.1182 to 0.01423	No	ns	0.1663
DIV10 vs. DIV12	–0.02767	–0.09390 to 0.03856	No	ns	0.673

One-way ANOVA followed by Post-hoc-Tukey's multiple comparison test; *n* refers to the analysis of PSD-95 and menin D.O.C. within different Days in-vitro (DIV) in hippocampal neuronal cultures, ROIs derived from 18 separate images from 12 independent experiments; ns ≥ 0.05

Supplementary Table S13b. Related to Figure 8. Mean of degree of colocalization of PSD-95 with menin in Days in-vitro (DIV) in hippocampal neuronal cultures.

Degree of colocalization	DIV4	DIV7	DIV10	DIV12
**Mean**	0.8857	0.8779	0.9022	0.9299
**Std. Error of Mean**	0.01658	0.02512	0.01543	0.007131

Supplementary Table S14a. Related to Figure 8. Mean comparison of degree of colocalization of SYT with menin between different Days in-vitro (DIV) in hippocampal neuronal cultures.

DIV	Mean Diff.	95.00% CI of diff.	Significant?	Summary	Adjusted *p* Value
DIV4 vs. DIV7	0.04113	–0.03678 to 0.1190	No	ns	0.4852
DIV4 vs. DIV10	–0.07525	–0.1532 to 0.002659	No	ns	0.0612
DIV4 vs. DIV12	–0.1363	–0.2142 to -0.05834	Yes	***	0.0003
DIV7 vs. DIV10	–0.1164	–0.1943 to -0.03847	Yes	**	0.0018
DIV7 vs. DIV12	–0.1774	–0.2553 to -0.09947	Yes	****	<0.0001
DIV10 vs. DIV12	–0.061	–0.1389 to 0.01691	No	ns	0.166

One-way ANOVA followed by Post-hoc-Tukey's multiple comparison test; *n* refers to the analysis of SYT and menin D.O.C. within different Days *in-vitro* (DIV) in hippocampal neuronal cultures, ROIs derived from 18 separate images from 12 independent experiments; ** $p < 0.01$, *** $p < 0.001$, **** $p < 0.0001$, ns ≥ 0.05

Supplementary Table S14b. Related to Figure 8. Mean of degree of colocalization of SYT with menin in Days in-vitro (DIV) in hippocampal neuronal cultures.

Degree of colocalization	DIV4	DIV7	DIV10	DIV12
**Mean**	0.7878	0.7466	0.863	0.924
**Std. Error of Mean**	0.03079	0.02226	0.009233	0.009979

Supplementary Table S15. Related to Figure 9. Mean of degree of colocalization of GABAB with menin in distinct regions of adult mouse brain.

De	Co	Hc	Th	Ce
**Mean**	0.646	0.49	0.618	0.45
**Std. Error of Mean**	0.01648	0.02011	0.01896	0.01282
**Test Statistics**	t = 8.860, df = 9	t = 0.4972, df = 9	t = 6.223, df = 9	t = 3.899, df = 9
***p* Value**	<0.0001	0.6309	0.0002	0.0036
**Summary**	****	ns	***	**

One sample t-test; *n* refers to the analysis of GABA B and menin D.O.C. within distinct regions of adult mouse brain, ROIs derived from 14 separate images from 8 independent experiments; ** $p < 0.01$, *** $p < 0.001$, **** $p < 0.0001$, ns ≥ 0.05

Supplementary Table S16. Related to Figure 9. Mean of degree of colocalization of mGluR1 with menin in distinct regions of adult mouse brain.

Degree of colocalization	Co	Hc	Th	Ce
**Mean**	0.885	0.795	0.884	0.81
**Std. Error of Mean**	0.01176	0.01204	0.01118	0.01135
**Test Statistics**	t = 24.50, df = 9	t = 32.73, df = 9	t = 34.36, df = 9	t = 27.31, df = 9
***p* Value**	<0.0001	<0.0001	<0.0001	<0.0001
**Summary**	****	****	****	****

One sample t-test; *n* refers to the analysis of GABA B and menin D.O.C. within distinct regions of adult mouse brain, ROIs derived from 14 separate images from 8 independent experiments; **** $p < 0.0001$

Supplementary Table S17. Related to Figure 9. Mean comparison of degree of colocalization of mGluR1 and GABAB with menin in distinct regions of adult mouse brain.

Treatment	Mean Diff.	95.00% CI of diff.	Significant?	Summary	Adjusted *p* Value
Co	–0.239	–0.2922 to –0.1858	Yes	****	<0.0001
Hc	–0.305	–0.3582 to –0.2518	Yes	****	<0.0001
Th	–0.266	–0.3192 to –0.2128	Yes	****	<0.0001
Ce	–0.36	–0.4132 to –0.3068	Yes	****	<0.0001

Two-way ANOVA followed by Sidak's multiple comparison test; *n* refers to the mean comparison of D.O.C. of mGluR1 of GABA B with menin within distinct regions of adult mouse brain, ROIs derived from 14 separate images from 8 independent experiments; **** $p < 0.0001$

Supplementary Table S18a. Related to Figure 9. Mean comparison of degree of colocalization of GABAA with menin between different aged mouse brain (temporal).

	E12.5	E15	E18	P10	Adult
**Actual mean**	0.7582	0.6555	0.5155	0.4700	0.9409
**One sample t test**					
**t, df**	t = 11.51, df = 10	t = 4.008, df = 10	t = 0.6602, df = 10	t = 1.318, df = 10	t = 0.8704, df = 10
***p* value (two tailed)**	<0.0001	0.0025	0.5240	0.2169	0.4045
***p* value summary**	****	**	ns	ns	ns
**Significant (alpha = 0.05)?**	Yes	Yes	No	No	No

One sample t-test; *n* refers to the analysis of GABA A and menin D.O.C. between different ages of mouse brain, ROIs derived from 14 separate images from 13 independent experiments; ** $p < 0.01$, **** $p < 0.0001$, ns ≥ 0.05

Supplementary Table S18b. Related to Figure 7. Mean comparison of degree of colocalization of GABAB with menin between different aged mouse brain (temporal).

	E12.5	E15	E18	P10	Adult
**Actual mean**	0.7691	0.7560	0.5015	0.4907	0.4686
**One sample t test**					
**t, df**	t = 9.002, df = 10	t = 10.64, df = 10	t = 0.05729, df = 10	t = 0.4312, df = 10	t = 0.8284, df = 10
***p* value (two tailed)**	<0.0001	<0.0001	0.9554	0.6755	0.4268
***p* value summary**	****	****	ns	ns	ns
**Significant (alpha = 0.05)?**	Yes	Yes	No	No	No

One sample t-test; *n* refers to the analysis of GABA B and menin D.O.C. between different ages of mouse brain, ROIs derived from 14 separate images from 13 independent experiments; **** $p < 0.0001$, ns ≥ 0.05

Supplementary Table S18c. Related to Figure 7. Mean comparison of degree of colocalization of mGluR1 with menin between different aged mouse brain (temporal).

	E12.5	E15	E18	P10	Adult
**Actual mean**	0.3665	0.5797	0.6801	0.8008	0.8643
**One sample t test**					
**t, df**	t = 2.891, df=10	t = 3.468, df = 10	t = 5.379, df = 10	t = 9.550, df = 10	t = 12.57, df = 10
***p* value (two tailed)**	0.0161	0.0060	0.0003	<0.0001	<0.0001
***p* value summary**	*	**	***	****	****
**Significant (alpha = 0.05)?**	Yes	Yes	Yes	Yes	Yes

One sample t-test; n refers to the analysis of mGluR1 and menin D.O.C. between different ages of mouse brain, ROIs derived from 14 separate images from 13 independent experiments; * $p < 0.1$, ** $p < 0.01$, *** $p < 0.001$, **** $p < 0.0001$

Supplementary Table S18d. Related to Figure 7. Mean comparison of degree of colocalization of GluR1 with menin between different aged mouse brain (temporal).

	E12.5	E15	E18	P10	Adult
**Actual mean**	0.3773	0.4970	0.6450	0.8233	0.9050
**One sample t test**					
**t, df**	t = 5.277, df = 9	t = 0.1103, df = 9	t = 5.564, df = 9	t = 11.90, df = 9	t = 13.68, df = 9
***p* value (two tailed)**	0.0005	0.9146	0.0004	<0.0001	<0.0001
***p* value summary**	***	ns	***	****	****
**Significant (alpha = 0.05)?**	Yes	No	Yes	Yes	Yes

One sample t-test; n refers to the analysis of GluR1 and menin D.O.C. between different ages of mouse brain, ROIs derived from 14 separate images from 13 independent experiments; *** $p < 0.001$, **** $p < 0.0001$, ns ≥ 0.05

Supplementary Table S18e. Related to Figure 7. Mean comparison of degree of colocalization of GABAB, GABAA, mGluR1 and GluR1 with menin between different aged mouse brain (temporal).

**Tukey's multiple comparisons test**	**95.00% CI of diff.**	**Significant?**	**Summary**	**Adjusted *p* Value**	**q**	**df**
**E12.5**						
GABA A vs. GluR1	0.2715 to 0.4903	Yes	****	<0.0001	12.76	195.0
GABA B vs. mGluR1	0.2959 to 0.5094	Yes	****	<0.0001	13.82	195.0
**E15**						
GABA A vs. GluR1	0.04900 to 0.2678	Yes	**	0.0013	5.306	195.0
GABA B vs. mGluR1	0.06949 to 0.2831	Yes	***	0.0002	6.050	195.0
**E18**						
GABA A vs. GluR1	−0.2389 to −0.02009	Yes	*	0.0131	4.338	195.0
GABA B vs. mGluR1	−0.2853 to −0.07179	Yes	***	0.0001	6.129	195.0
**P10**						
GABA A vs. GluR1	−0.4627 to −0.2439	Yes	****	<0.0001	11.83	195.0

GABA B vs. mGluR1	–0.4169 to –0.2033	Yes	****	<0.0001	10.64	195.0
**Adult**						
GABA A vs. GluR1	–0.5644 to –0.3456	Yes	****	<0.0001	15.24	195.0
GABA B vs. mGluR1	–0.5025 to –0.2889	Yes	****	<0.0001	13.58	195.0

One-way ANOVA followed by Post-hoc-Tukey's multiple comparison test; *n* refers to the analysis of GABAA, GABAB, mGluR1, GluR1 and menin D.O.C. within different aged whole brain slices, ROIs derived from 14 separate images from 13 independent experiments; * $p < 0.1$, ** $p < 0.01$, *** $p < 0.001$, **** $p < 0.0001$

Supplementary Table S19a. Related to Figure 9. Mean of degree of colocalization of α7 nAChRs with menin in distinct regions of adult mouse brain.

Degree of colocalization	Co	Hc	Th	Ce
**Mean**	0.861	0.914	0.868	0.856
**Std. Error of Mean**	0.013699	0.004	0.021695	0.01376
**Test Statistics**	t = 103.5, df = 9	t = 26.35, df = 9	t = 16.96, df = 9	t = 25.87, df = 9
***p* Value**	<0.0001	<0.0001	<0.0001	<0.0001
**Summary**	****	****	****	****

One sample t-test; *n* refers to the analysis of α7 nAChRs and menin D.O.C. within distinct regions of adult mouse brain, ROIs derived from 15 separate images from 6 independent experiments; **** $p < 0.0001$

Supplementary Table S19b. Related to Figure 9. Mean of degree of colocalization of α5 nAChRs with menin in distinct regions of adult mouse brain.

Degree of colocalization	Co	Hc	Th	Ce
**Mean**	0.857	0.888	0.8992	0.873
**Std. Error of Mean**	0.024224	0.013317	0.015003	0.010651
**Test Statistics**	t = 29.14, df = 9	t = 14.74, df = 9	t = 26.61, df = 9	t = 35.02, df = 9
***p* Value**	<0.0001	<0.0001	<0.0001	<0.0001
**Summary**	****	****	****	****

One sample t-test; *n* refers to the analysis of α5 nAChRs and menin D.O.C. within distinct regions of adult mouse brain, ROIs derived from 15 separate images from 9 independent experiments; **** $p < 0.0001$

Supplementary Table S19c. Related to Figure 9. Mean of degree of colocalization of α7 nAChRs with menin in different ages of whole mouse brain slices.

Degree colocalization	E15	E18	P10	Adult
**Mean**	0.7663	0.8492	0.8685	0.9143
**Std. Error of Mean**	0.007174	0.006198	0.01425	0.01108

Supplementary Table S19d. Related to Figure 9. Mean comparison of degree of colocalization of α7 nAChRs with menin between different aged mouse brain slices (temporal).

Ages	Mean Diff.	95.00% CI of diff.	Significant?	Summary	Adjusted *p* Value
E15 vs. E18	–0.0829	–0.1217 to -–0.04407	Yes	****	<0.0001
E15 vs. P10	–0.1022	–0.1410 to –0.06333	Yes	****	<0.0001
E15 vs. Adult	–0.148	–0.1868 to –0.1092	Yes	****	<0.0001
E18 vs. P10	–0.01926	–0.05809 to 0.01957	No	ns	0.5467
E18 vs. Adult	–0.0651	–0.1039 to –0.02627	Yes	***	0.0004
P10 vs. Adult	–0.04584	–0.08467 to –0.007013	Yes	*	0.0153

One-way ANOVA followed by Post-hoc-Tukey's multiple comparison test; *n* refers to the analysis of α7 nAChRs and menin D.O.C. within different aged whole brain slices, ROIs derived from 15 separate images from 9 independent experiments; * $p < 0.1$,*** $p < 0.001$, **** $p < 0.0001$, ns ≥ 0.05

Supplementary Table S19e. Related to Figure 9. Mean of degree of colocalization of α5 nAChRs with menin in different ages of whole mouse brain slices.

Fluorescence Intensity	E15	E18	P10	Adult
**Mean**	0.832	0.8818	0.9288	0.9343
**Std. Error of Mean**	0.00611	0.00627	0.004765	0.007059

Supplementary Table S19f. Related to Figure 9. Mean comparison of degree of colocalization of α5 nAChRs with menin between different aged mouse brain slices (temporal).

**Ages**	**Mean Diff.**	**95.00% CI of diff.**	**Significant?**	**Summary**	**Adjusted *p* Value**
E15 vs. E18	–0.0498	–0.07306 to –0.02654	Yes	****	<0.0001
E15 vs. P10	–0.0968	–0.1201 to –0.07354	Yes	****	<0.0001
E15 vs. Adult	–0.1023	–0.1256 to –0.07904	Yes	****	<0.0001
E18 vs. P10	–0.047	–0.07026 to -–0.02374	Yes	****	<0.0001
E18 vs. Adult	–0.0525	–0.07576 to –0.02924	Yes	****	<0.0001
P10 vs. Adult	–0.0055	–0.02876 to 0.01776	No	ns	0.9194

One-way ANOVA followed by Post-hoc-Tukey's multiple comparison test; n refers to the analysis of α7 nAChRs and menin D.O.C. within different aged whole brain slices, ROIs derived from 15 separate images from 9 independent experiments; **** $p < 0.0001$, ns ≥ 0.05

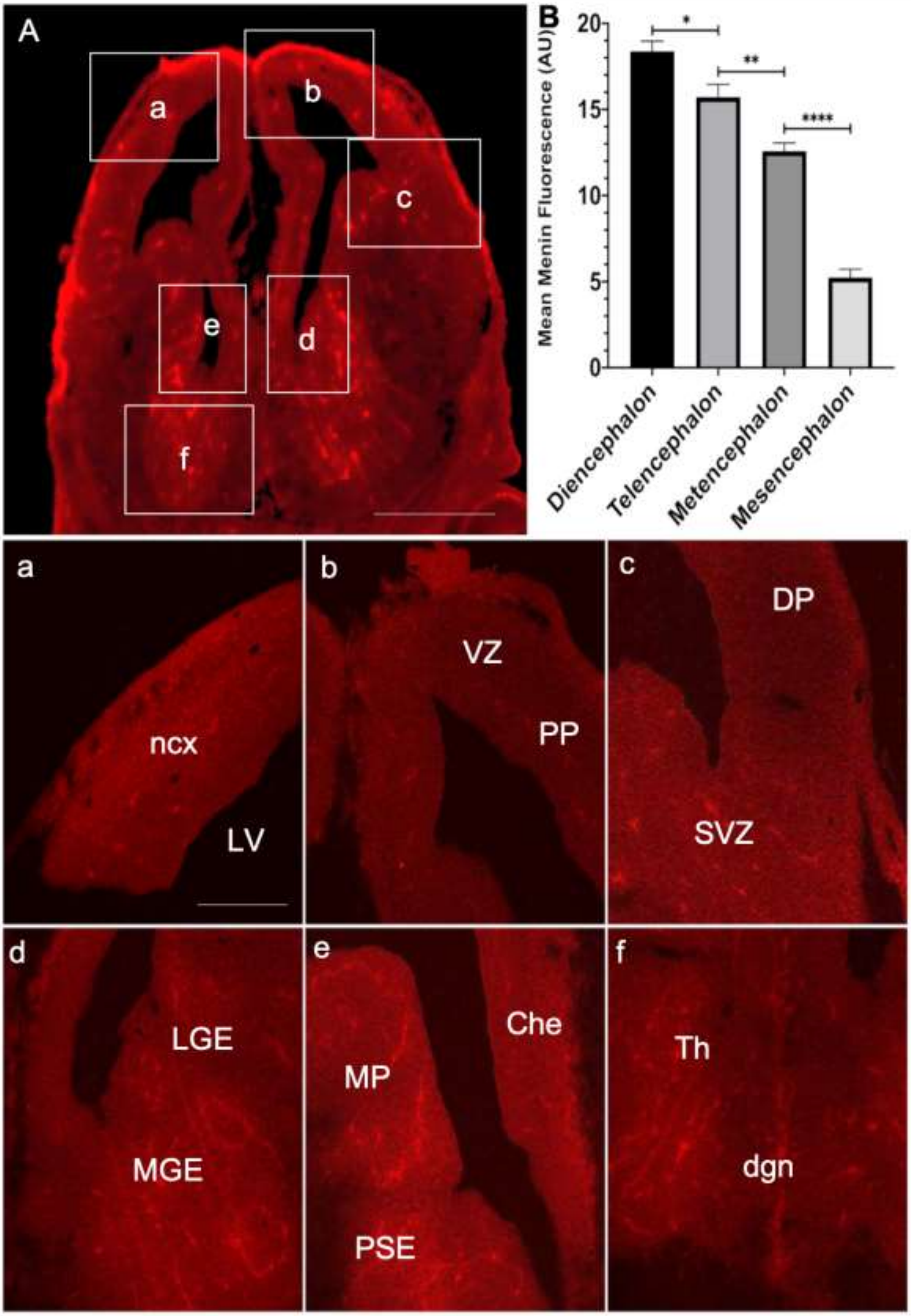

Supplementary Figure S1. Related to Figure 5. Menin protein expression in E12.5 embryonic mouse brain. (**A**). Menin protein distribution is represented on coronal sections in a rostro-caudal sequence (panels **a**–**f**). IHC characterization of menin protein at E12.5 shows high localization in the telencephalon especially in lateral and medial ganglionic eminence (panels **d**) in the neocortex (panel **a**) and in the subventricular zone (panel **c**). The ventral thalamus (panel **e** and **f**) and hypothalamus (panel **c**) showed strongest immunoreactivity with menin protein. Arrowheads indicate high localization of menin protein. Scale bar: (**A**)250 μm, panels **a**–**f** 100 μm (**B**). Summary data, normalized fluorescence intensity of α-C-

terminal menin in different regions of E12.5 mouse brain (ROIs: n = 13 each from seven independent samples; see Table S1a and S1b). Asterisks, statistical significance (One-way ANOVA followed by post-hoc tukey test); * $p < 0.05$. ** $p < 0.01$.**** $p < 0.0001$. **Abbreviations**: ncx: neocortex, LV: Lateral ventricle, VZ: ventral zone, PP: preplate, DP:dorsal plate, SVZ: subventricular zone, LGE: lateral ganglionic eminence, MGE: medial ganglionic eminence, MP: medioposterior nucleus, Che: choroid plexus, PSE: paraseptal subpallial sector, Th: thalamus, dgn: Dentate gyrus neuroepithelium.

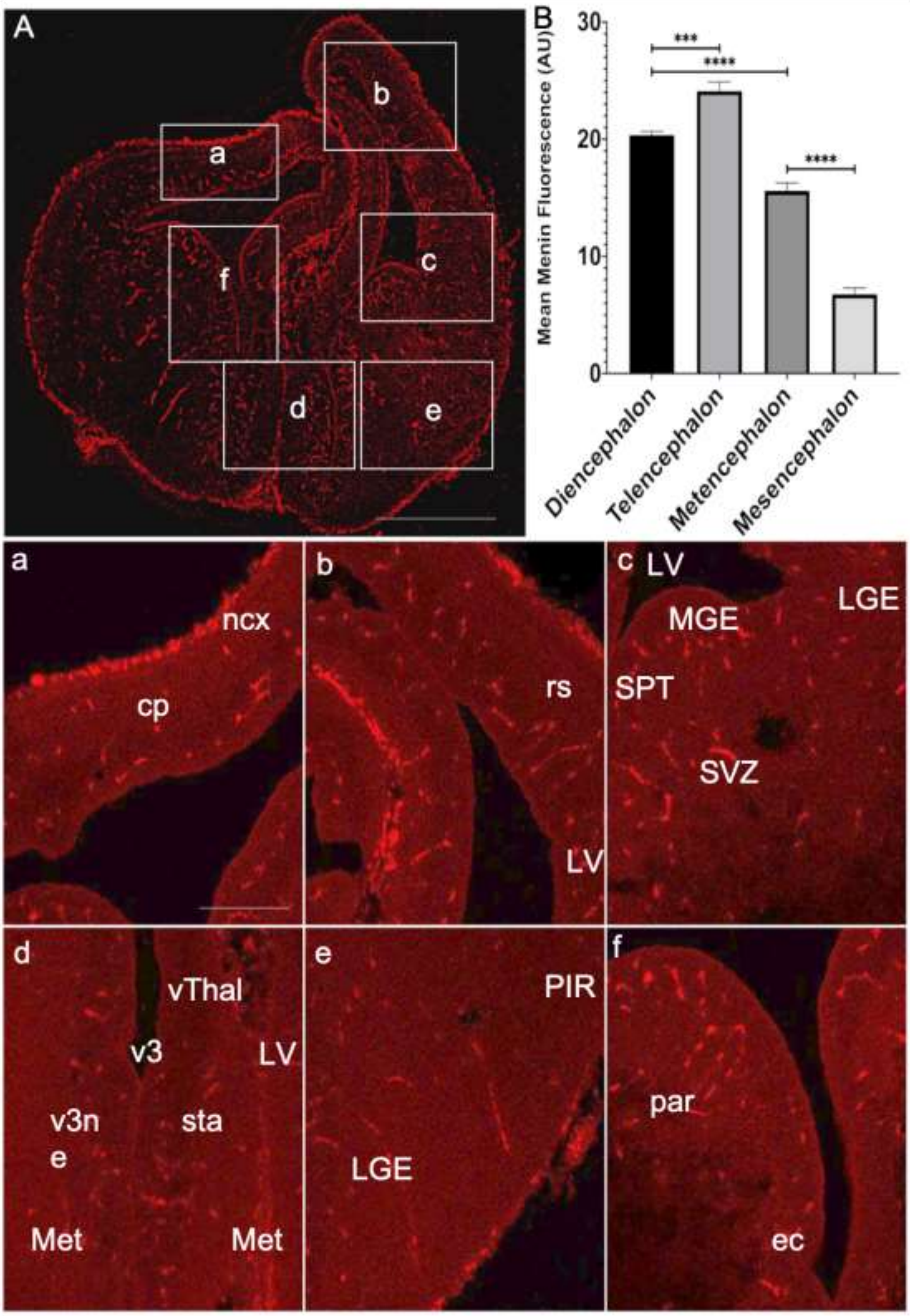

Supplementary Figure S2. Related to Figure 5. Menin protein expression in E15.5 embryonic mouse brain. (**A**). Menin protein distribution is represented on coronal sections in a rostro-caudal sequence (panels **a**–**f**). IHC characterization of menin protein at E15.5 displays highest localization in the telencephalic structures specifically in neocortex, retro-splenial

cortex, lateral ventricle (panel **a,b**) whereas moderate to low menin expression in septum, subventricular zone, external capsule and parietal cortex (panel **c,e**). The ventral thalamus, regions around the third ventricle, subthalamic area (panel d) also showed strong immunoreactivity with menin protein. Arrowheads indicate high localization of menin protein. Scale bar: (**A**) 250 µm, panels (a–f) 100 µm (**B**). Summary data, normalized fluorescence intensity of α-C-terminal menin in different regions of E15.5 mouse brain (ROIs: n = 13 each from seven independent samples; see Table S2a and S2b). Asterisks, statistical significance (One-way ANOVA followed by post-hoc tukey test); *** $p < 0.001$. **** $p < 0.0001$. **Abbreviations**: ncx: neocortex, Cp: cortical plate, rs: retrosplenial cortex, LV: Lateral ventricle, VZ: ventral zone, SVZ: subventricular zone, LGE: lateral ganglionic eminence, MGE: medial ganglionic eminence, SPT: septum, v3: third ventricle, V3ne: third ventricle neuroepithelium, sta: striatum, Met: mesencephalic tegmentum, PIR: piriform cortex, par: Parietal cortex, ec: External capsule, vThal: ventral thalamus.

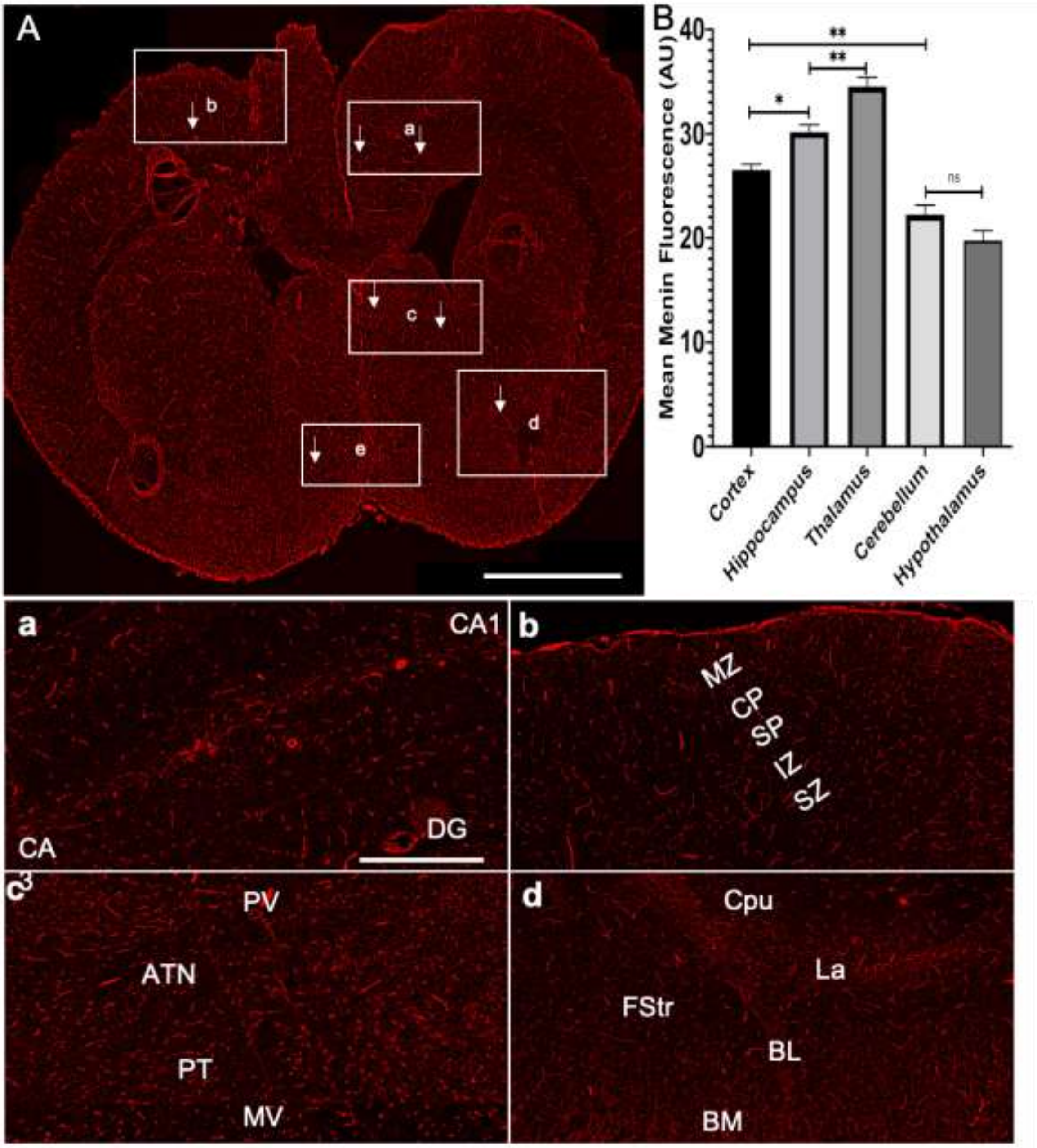

Supplementary Figure S3. Related to Figure 5. Menin protein expression in E18 embryonic mouse brain. (**Aa–e**). Menin protein distribution is represented on coronal sections in a rostro-caudal sequence (panels **a**–**d**). IHC characterization of menin protein at E18 demonstrates high localization in the telencephalon, hippocampus and cortex exhibited significantly robust localization of menin protein, with highest expression being in the dentate gyrus and CA1 regions of the hippocampus (panel a), in neocortex, frontal cortex, cingulate cortex (panel b) and moderate expression in parietal cortex and insular cortex (panel d). Within the diencephalon and thalamus exhibiting strong and ubiquitous signal, specifically in the nuclei of dorsal and ventral thalamic regions (panel c). Scale bar: (**A**)300 µm, panels (**a**–**d**) 150 µm (**B**). Summary data, normalized fluorescence intensity of α-C-terminal menin in different regions of E12.5 mouse brain (ROIs: n = 13 each from

seven independent samples; see Table S3a and S3b). Asterisks, statistical significance (One-way ANOVA followed by post-hoc tukey test); * $p < 0.05$. ** $p < 0.01$, ns $p > 0.05$. **Abbreviations**: CA1: Cornu Ammonis-1, CA3 Cornu Ammonis-3, DG: Dentate gyru, MZ: marginal zone, CP: cortical plate, SP: Subplate, IZ: Intermediate zone, SZ: subventricular zone, PV: paraventricular, ATN: anterior thalamic nuclei, PT: pretectum, MV: medioventral, Cpu: Caudate putamen, La: Lateral nucleus of amygdala, BL: Basolateral amygdaloid nucleus, FStr: Fundus Striati, BM: Basomedial amygdaloid nucleus.

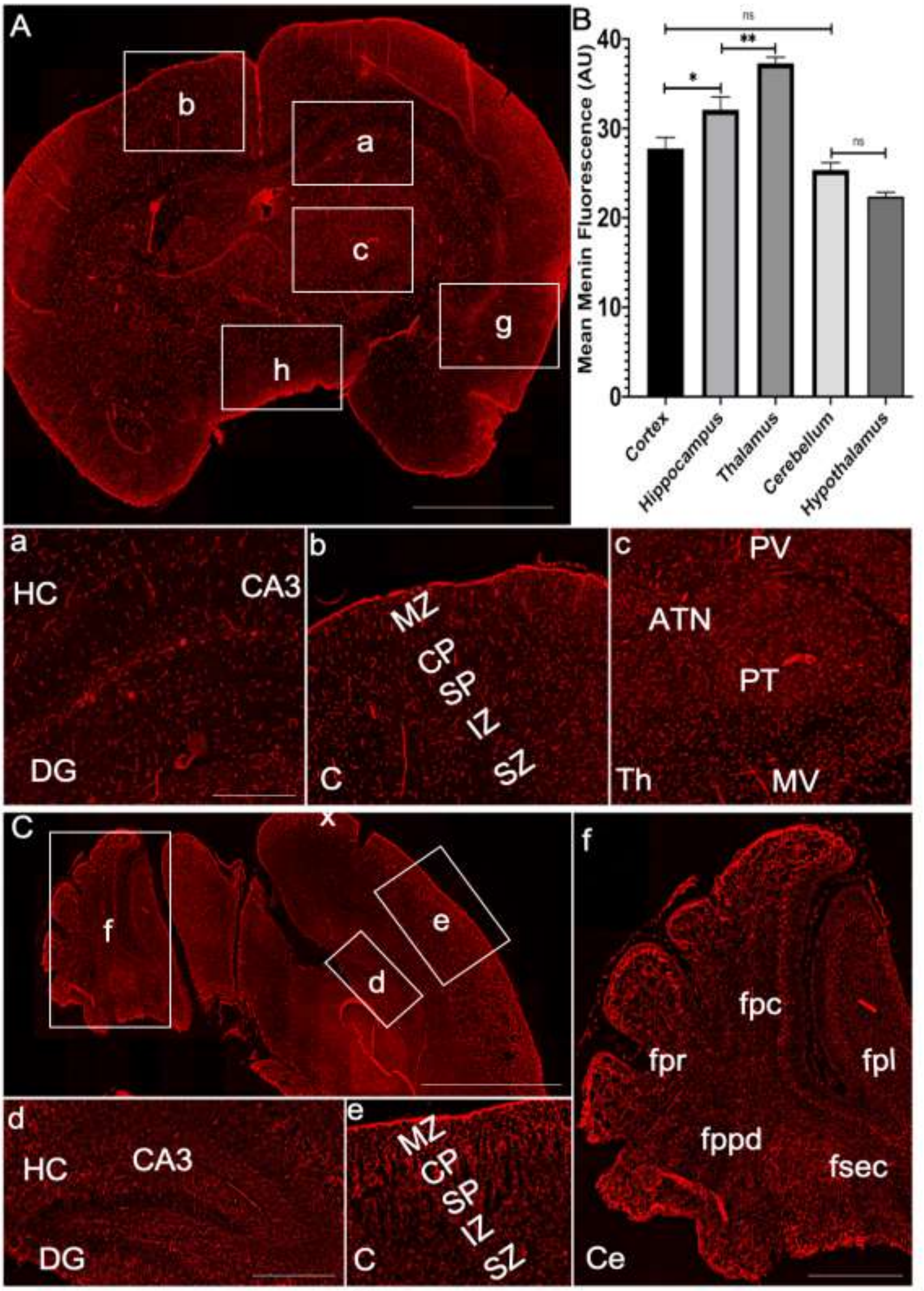

Supplementary Figure S4. Related to Figure 5. Menin protein expression in P10 mouse brain. (**A**,**B**). Menin protein distribution is represented on coronal sections in a rostro-caudal sequence (panels **a**–**c**,**g**,**h**) and on a sagittal section (panels **d**,**e**,**f**). IHC characterization of menin protein at P10 shows high localization in the telencephalon especially strong

expression in the dentate gyrus, CA1 and CA3 regions in hippocampus (panel **a,d**), in neocortex, frontal cortex, cingulate cortex, subventricular zone (panel **b,e**) and low expression in parietal cortex and insular cortex (panel **g**). Within the diencephalon, high localization in nuclei of dorsal, central and ventral thalamic regions (panel **c**). Whereas moderate menin fluorescence intensity was observed in hypothalamus (panel **h**). Within the metencephalon, cerebellum displayed strong fluorescence signal in the p10 mouse brain (panel **f**). Scale bar: (**A**).350 µm, panels (**a–c**) 75 µm, panel (d) 70 µm, panel (**e**) 65 µm, panels (**f**) 100 µm (**B**) 300 µm.(**C**). Summary data, normalized fluorescence intensity of α-C-terminal menin in different regions of P10 mouse brain (ROIs: n = 13 each from seven independent samples; see Table S4a and S4b). Asterisks, statistical significance (One-way ANOVA followed by post-hoc tukey test); * $p < 0.05$. ** $p < 0.01$, ns > 0.05 **Abbreviations**: HC: hippocampus, CA1: Cornu Ammonis-1, CA3 Cornu Ammonis-3, DG: Dentate gyru, C: cortex, MZ: marginal zone, CP: cortical plate, SP: Subplate, IZ: Intermediate zone, SZ: subventricular zone, PV: paraventricular, Th: thalamus, ATN: anterior thalamic nuclei, PT: pretectum, MV: medioventral, Cpu: Caudate putamen, La: Lateral nucleus of amygdala, BL: Basolateral amygdaloid nucleus,Ce, cerebellum, FSec: secondary fissure; Fpl: posterolateral fissure, fppd: prepyramidal fissure, fpc: preculminate fissure.

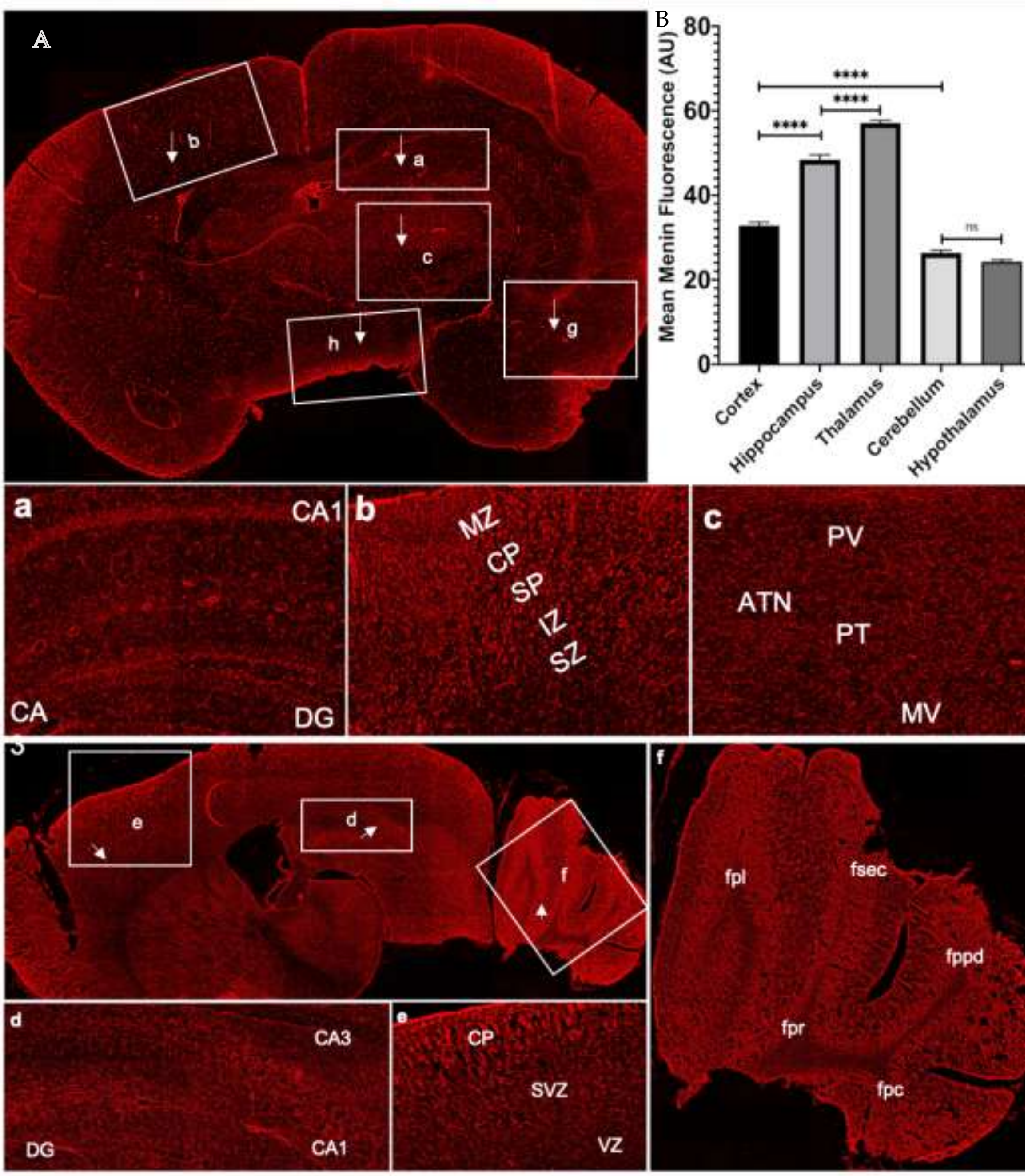

Supplementary Figure S5. Related to Figure 5. Menin protein expression in an adult mouse brain. (**A,B**). Menin protein distribution is represented on coronal sections in a rostro-caudal sequence (panels **a**–**c**,**g**,**h**) and on a sagittal section (panels **d,e,f**). IHC characterization of menin protein in an adult mouse brain slice exhibits high localization in the telencephalon especially in in dentate gyrus, CA1, CA2 and CA3 regions in hippocampus (panel **a,d**), in neocortex, frontal cortex,

cingulate cortex, subventricular zone (panel **b,e**) and low localization in parietal cortex and insular cortex (panel **g**). Within the diencephalon, high localization in nuclei of dorsal, central and ventral thalamic regions (panel **c**), whereby moderate expression was observed in the hypothalamus region (panel **h**). Within the metencephalon, cerebellum exhibited high menin fluorescence intensity in the adult mouse brain (panel **f**). Scale bar: (**A**).350 μm, panels (**a–c**) 75 μm, panel (**d**) 70 μm, panel (**e**) 65 μm, panels (**f**) 100 μm (**B**) 300 μm. (**B**) 300 μm.(**B**). Summary data, normalized fluorescence intensity of α-C-terminal menin in different regions of adult mouse brain (ROIs: n = 13 each from 7 independent samples; see Supplementary Table S51a,b). Asterisks, statistical significance (One-way ANOVA followed by post-hoc tukey test);. **** $p < 0.0001$, ns >0.05 **Abbreviations**: HC: hippocampus, CA1: Cornu Ammonis-1, CA3: Cornu Ammonis-3, DG: Dentate gyru, C:cortex, MZ: marginal zone, CP: cortical plate, SP: Subplate, IZ: Intermediate zone, SZ: subventricular zone, PV: paraventricular , Th:thalamus,ATN: anterior thalamic nuclei,PT: pretectum,MV: medioventral,Cpu: Caudate putamen,La: Lateral nucleus of amygdala, BL: Basolateral amygdaloid nucleus, Ce cerebellum, FSec: secondary fissure, Fpl: posterolateral fissure, fppd: prepyramidal fissure, fpc: preculminate fissure.

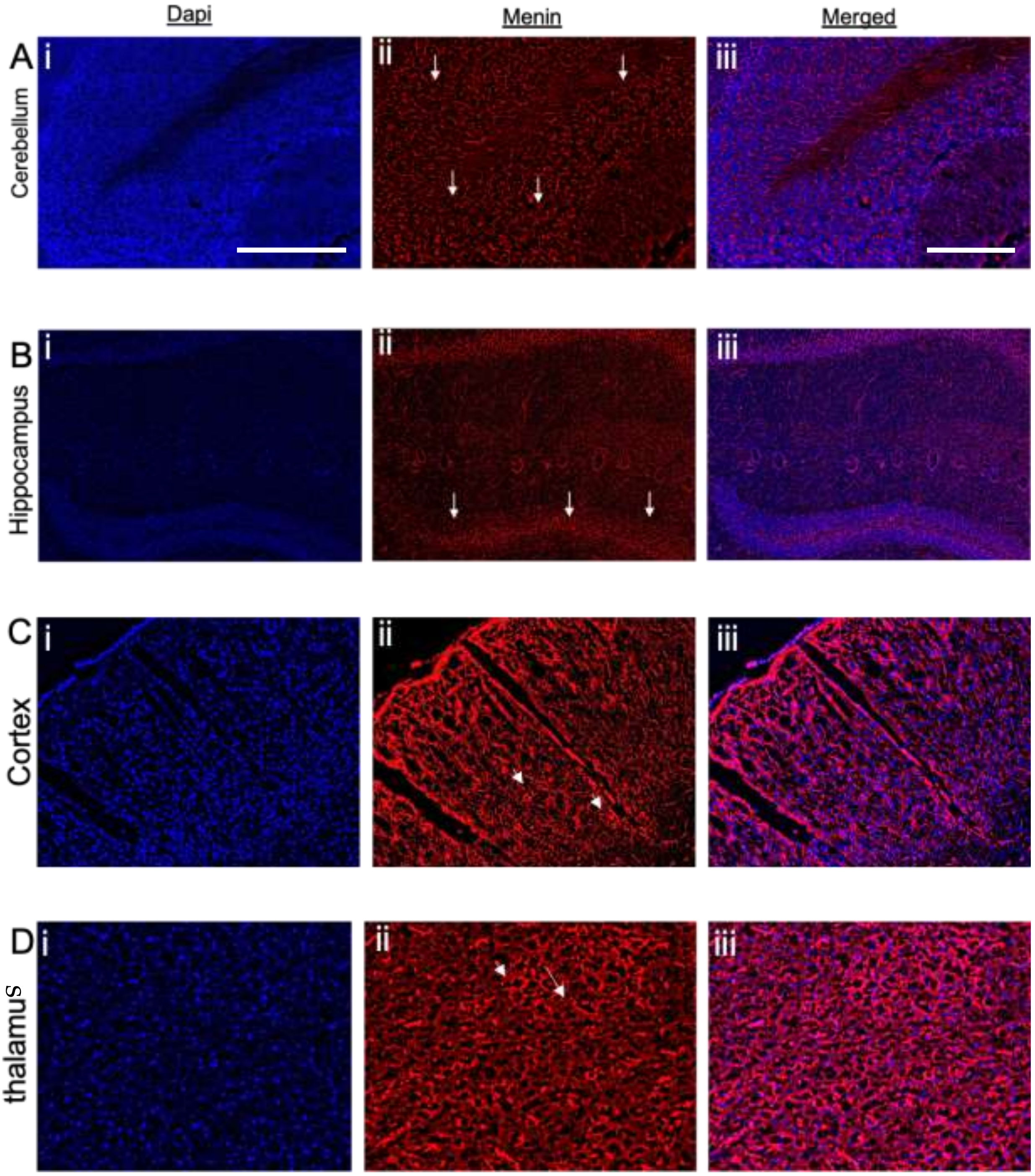

Supplementary Figure S6. Related to Figure 6. Menin exhibits neuron-specific expression in specific regions of brain. (**Ai–Diii**). IHC localization of menin in cerebellum, hippocampus, cortex and thalamus brain slice of adult mouse brain

(representative images). (**A–Di**). Slices labeled with DAPI for nuclear stain (**A–Dii**). Slices labeled with C-terminal epitope menin antibody. (**A–Diii**). Slice shows merged channels C-terminal menin with the nuclear stain DAPI. (n=24 images, 5 independent samples). Asterisks represent α-C-menin's distinct localization in cytoplasm also punctuate localization along neurites indicated by arrowheads. Scale bars **A–Di,ii**: 75μm, **A–Diii**:100 μm.

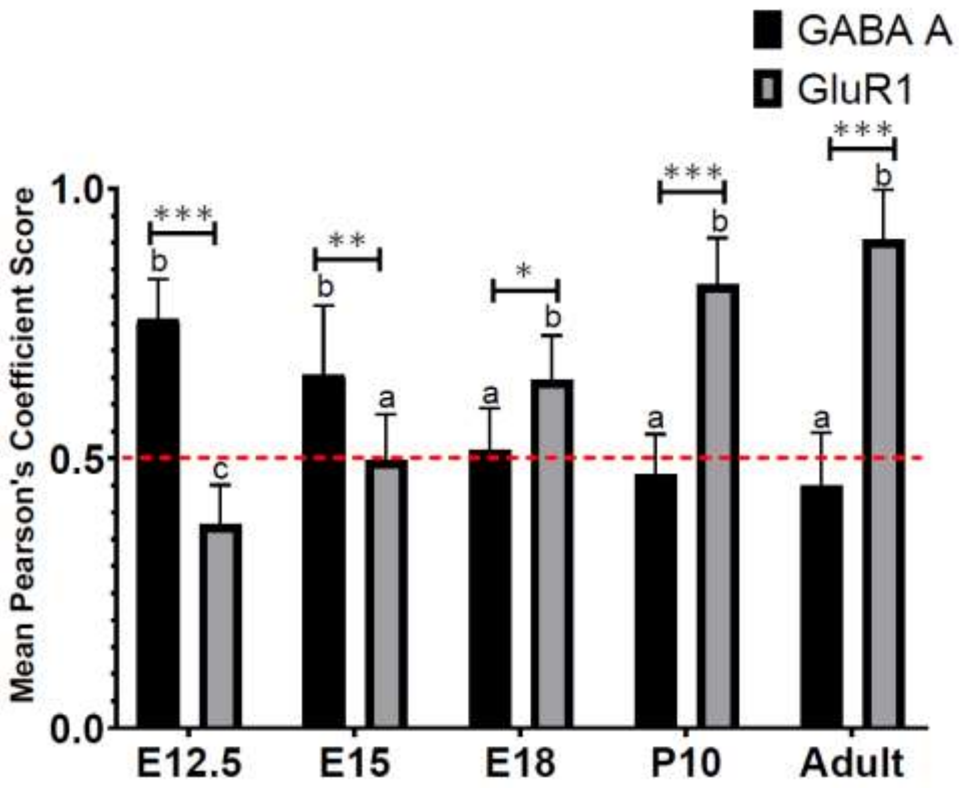

Supplementary Figure S7. Related to Figures 9. Summary data, Pearson's co-efficient, temporal pattern degree of colocalization of GABA A and GluR1 puncta with C-menin in E12.5, E15.5, E18, P10 and adult mouse brain mouse brain. Red dotted line indicates the literature value of significant DOC; group a (DOC significant precisely at 0.5), b (DOC significant) and group c (no significant DOC) (One sample t-test). Asterisks, statistical significance (One-way ANOVA followed by Sidak's comparison test); * p <0.1. ** $p < 0.01$. *** $p < 0.001$.
